# AKT and SGK kinases regulate cell migration by altering Scar/WAVE complex activation and Arp2/3 complex recruitment

**DOI:** 10.3389/fmolb.2022.965921

**Published:** 2022-08-29

**Authors:** Shashi Prakash Singh, Peggy Paschke, Luke Tweedy, Robert H. Insall

**Affiliations:** ^1^ CRUK Beatson Institute, Glasgow, United Kingdom; ^2^ School of Infection and Immunity, University of Glasgow, Glasgow, United Kingdom; ^3^ School of Cancer Sciences, University of Glasgow, Glasgow, United Kingdom

**Keywords:** cell motility, pkbA, pkgB, actin, Scar/WAVE complex, Arp2/3 complex

## Abstract

Cell polarity and cell migration both depend on pseudopodia and lamellipodia formation. These are regulated by coordinated signaling acting through G-protein coupled receptors and kinases such as PKB/AKT and SGK, as well as the actin cytoskeletal machinery. Here we show that both *Dictyostelium* PKB and SGK kinases (encoded by *pkbA* and *pkgB*) are dispensable for chemotaxis towards folate. However, both are involved in the regulation of pseudopod formation and thus cell motility. Cells lacking *pkbA* and *pkgB* showed a substantial drop in cell speed. Actin polymerization is perturbed in *pkbA-* and reduced in *pkgB-* and *pkbA-/pkgB-* mutants. The Scar/WAVE complex, key catalyst of pseudopod formation, is recruited normally to the fronts of all mutant cells (*pkbA*-, *pkgB*- and *pkbA*-/*pkgB*-), but is unexpectedly unable to recruit the Arp2/3 complex in cells lacking SGK. Consequently, loss of SGK causes a near-complete loss of normal actin pseudopodia, though this can be rescued by overexpression of PKB. Hence both PKB and SGK are required for correct assembly of F-actin and recruitment of the Arp2/3 complex by the Scar/WAVE complex during pseudopodia formation.

## Introduction

Cell migration is a fundamental biological process required for immune cell function, development and differentiation, among other roles. Alterations in migration can play central roles in diseases like cancer (Talmadge and Fidler, 2010; Bravo-Cordero et al., 2012) and infections (Zaki et al., 2006). Cell migration can be random or directional. Directed migration of cells is highly orchestrated and can occur in response to external chemical cues or signaling (Affolter and Weijer, 2005; Wang et al., 2011; Artemenko et al., 2014) called chemotaxis.

Cells move by forming actin rich structures, such as lamellipodia and pseudopodia. These protrusive structures are made through actin polymerization. Actin branching is mediated by the actin related protein complex Arp2/3, whose activation is driven ubiquitously by the recruitment of the Scar/WAVE complex at the front of the cells (Machesky and Insall, 1998; Krause and Gautreau, 2014; Schaks et al., 2019). The Scar/WAVE complex is an approximately 450 kDa hetero-pentameric complex, whose members are Pir121 (Cyfip), Nap1 (NCKAP1), Scar (WAVE), Abi, and HSPC300.

Activation of the Scar/WAVE complex at the leading edge is an essential step for the formation of pseudopodia, that is mediated by GTP bound active Rac1. At the cell front and in pseudopods, active Rac1 binds to the Pir121 subunit of the complex and activates it (Chen et al., 2010; Chen et al., 2017; Schaks et al., 2018). In addition to Rac1, phosphorylation of Scar/WAVE has been described as an additional regulator of the complex (Danson et al., 2007; Lebensohn and Kirschner, 2009; Mendoza et al., 2011). Earlier work flagged ERK2 kinase as important (Danson et al., 2007; Lebensohn and Kirschner, 2009; Mendoza et al., 2011), but we have recently shown that the SepA kinase, and not ERK2, phosphorylates *Dictyostelium* Scar/WAVE. However, neither controls its activation or inactivation. Still, phosphorylation of Scar/WAVE is useful as an indicator of the active state of the Scar/WAVE complex because it occurs immediately after the complex is activated (Singh et al., 2020; Singh et al., 2021). Phosphorylation of Scar/WAVE tunes cell migration by controlling the Scar/WAVE complex’s stability, lifetime, and recruitment to pseudopodia.

During our screen for kinases that phosphorylate Scar/WAVE, we also identified the *Dictyostelium* protein kinase B (PKB)/AKT homolog encoded by *pkbA* and the SGK kinase PkgB (also called PKB-R1) as potential interactors of the Scar/WAVE complex in protein crosslinking and pulldown experiments (Fort et al., 2018; Singh et al., 2020). Both proteins have been implicated in the regulation of actin-dependent processes as endocytosis (Williams et al., 2019) and chemotaxis (Meili et al., 1999; Meili et al., 2000; Kamimura et al., 2008; Kamimura and Devreotes, 2010), but their importance and their crosstalk with the Scar/WAVE complex are not yet understood.

The role of actin in macropinocytosis—in which cells feed by large scale uptake of liquid medium (Lewis, 1931; King and Kay, 2019)—is becoming clearer. Recruitment of the Scar/WAVE complex to macropinocytic cups is associated with F-actin polymerization (Veltman et al., 2016). Cells lacking Scar/WAVE complex members show defects in macropinocytosis. Similarly, cells lacking both PKB and PkgB show substantially reduced levels of macropinocytosis (Williams et al., 2019).

A range of publications describes the importance of PKB and PkgB to *Dictyostelium* directed cell migration and chemotaxis (Meili et al., 1999; Meili et al., 2000; Kamimura et al., 2008; Kamimura and Devreotes, 2010). Both kinases have been shown to be activated in response to cAMP (Meili et al., 1999; Meili et al., 2000), and PKB and PkgB phosphorylate common substrates after stimulation with cAMP or folate (Liao et al., 2010). These result in changes of the cytoskeleton during cell migration (Srinivasan et al., 2013). Cells lacking *pkbA* showed cell polarity defects and impaired motility (Meili et al., 1999), but only subtle migration defects were seen in cells lacking *pkgB* (Meili et al., 2000). In contrast, another report describes that cells lacking *pkgB* but not *pkbA* manifest severe defects in gradient sensing and chemotaxis (Kamimura et al., 2008). The chemotactic defects become even more severe in the *pkbA-/pkgB-* double knockout cells, and result in developmental arrest at the aggregation stage (Meili et al., 2000).

Altogether, this suggests that both kinases have roles in the regulation of cell motility and actin dynamics, though few mechanistic details are understood. In this study, we describe a link between Scar/WAVE phosphorylation, activation and both PKB, and PkgB kinases.

## Materials and methods

### Cell lines

All the cell lines Ax2, *pkbA-*, *pkgB*-, *pkbA*-/*pkgB*- used in this study were kind gifts from Dr. Robert R. Kay (Williams et al., 2019).

### *Dictyostelium* cell culture

All cell lines were grown with bacteria *Klebsiella aerogenes* on SM agar plates. Bacterial lawns were prepared on SM agar plates by spreading bacteria and allowing them to grow overnight at 22°C. Cells were streaked out on bacterial lawns with a sterile inoculation loop for approximately 48 h, by which time cells have cleared areas of bacteria and formed growth zones. Cells from growth zones were collected with a sterile inoculation loop (10 µL), washed 3 times with development buffer (DB) to clean remaining bacteria and used in under agarose chemotaxis or western blotting analyses.

### Plasmid constructs

Lifeact-mRFPmars2, mRFPmars2-ArpC4 or Pak-CRIB-mRFPmars2 expression fragments were cloned into pDM1091 using the NgoMIV restriction site to generate co expression constructs with HSPC300-eGFP. To construct extrachromosomal expression vectors of PKB and PkgB, *pkbA* and *pkgB* were PCR-amplified using Ax3 genomic DNA. *pkbA* was amplified using a forward primer (AGA​TCT​AAA​ATG​GGA​AAA​GGA​CAA​AGT​AA) and reverse primer (ACT​AGT​TAA​TCC​TTT​AAG​ATT​GAA​TCA​GC). *pkgB* was amplified using a forward primer (AGA​TCT​AAA​ATG​TCA​ACA​GCA​CCA​ATT​AAA​C) and reverse primer (ACT​AGT​TAT​CTT​AAA​TGT​TCA​GAT​TCA​GCG). Amplified products were cloned into pDM1033 using BglII and SpeI restriction sites.

### Transfection

Cells were grown on SM agar plates in conjunction with *Klebsiella aerogenes*. Cells were collected from the clearing zones with sterile inoculation loops and transfected as described in Paschke et al., 2019 (Paschke et al., 2019).

### Under agarose chemotaxis

Cellular morphology, pseudopod dynamics and cell migration were measured by an under-agarose folate chemotaxis assay as described previously (Singh et al., 2020; Singh and Insall, 2022). In brief, 0.4% SeamKem GTG agarose in Lo-Flo medium (Formedium) was boiled. After cooling, folic acid to a final concentration of 10 μM was added. 5 ml of agarose-folate mix was poured into the 1% BSA-coated 50 mm glass bottom dishes (MatTek). A 5 mm wide well was cut with a sterile scalpel and filled with 200 μL of 2 × 10^6^ cells/ml. Cell migration was imaged after 4–6 h with 10x and 60x differential interference contrast (DIC) microscopy. To examine the localization of labelled proteins in the pseudopods, cells were also imaged by AiryScan confocal microscopy.

### Western blotting

Cells were lysed by directly adding NuPAGE LDS sample buffer (Invitrogen) containing 20 mM DTT, HALT protease and phosphatase inhibitors (Thermo Fisher Scientific) onto the cells. Samples were boiled at 100°C for 5 min. Proteins were separated on 10% Bis-Tris NuPAGE gels (Invitrogen) or on hand-poured low-bis acrylamide (0.06% bis acrylamide and 10% acrylamide) gels, then separated at 150 V, 90 min. Proteins were transferred onto 0.45 μM nitrocellulose membrane. Membranes were blocked in TBS+5% non-fat milk. Primary antibodies, rabbit anti-Scar (Blagg et al., 2003), Abi (Pollitt and Insall, 2008), Nap1 and sheep anti-Pir121 (Ibarra et al., 2006), rat anti-GFP(3H9, Chromotek, RRID; AB_10773374) were used at 1:1000 dilution. Goat anti rabbit IgG (H + L) Dylight 800(Invitrogen; SA5-35571; RRID:AB_2556775) Rabbit anti Sheep IgG (H + L) Dylight 800 (Invitrogen, SA5-10058; RRID:AB_2556638) or Goat anti-Rat IgG (H + L) Dylight 800 (Invitrogen, SA5-10024; RRID:AB_2556604) fluorescently conjugated secondary antibodies at 1:10,000 dilution (were used to detect the protein bands using an Odyssey CLx Imaging system (LI-COR Biosciences). Mccc1 was used as a loading control (Davidson et al., 2013a). Streptavidin Alexa 680 conjugate was used to probe Mccc1 at 1:1000 dilution (Invitrogen, S32358).

### Phosphatase treatment

Cells grown in a 35 mm Petri dish were lysed in 100 μL TN/T buffer (10 mM Tris-HCl pH 7.5, 150 mM NaCl and 0.1% Triton X-100), kept on ice for 5 min and cleared by centrifugation (13,000 rpm, 4°C, 5 min). Proteins were dephosphorylated using 1 μL Lambda phosphatase at 30°C (NEB; P0753S) for 1 h. Protein dephosphorylation was assessed by western blotting.

### Microscopy

To determine the morphology, chemotactic speed and directionality of cells, phase contrast time lapse microscopy was performed at 10x/0.3NA on a Nikon ECLIPSE TE-2000-R inverted microscope equipped with a Retiga EXI CCD monochromatic camera. Images of cells migrating under agarose in a folate gradient were captured per minute for 45 min. DIC images were taken every 3 s for 3 min with 60x/1.4 NA to observe the pseudopod formation. HSPC300-eGFP expressing cells were used to determine the activation of the Scar/WAVE complex. Localization of the Scar/WAVE complex, Arp2/3 complex, and F- actin was examined by a 63x/1.4 NA objective on an AiryScan Zeiss 880 inverted confocal microscope. The brightness and contrast of the images were adjusted to visualize the cell protrusions and defects using ImageJ.

### Quantification of data and statistics

Each experiment was performed at least thrice. Speed of cells, chemotaxis efficiency index, directedness, circularity, cell area were calculated from the movies of cells migrating under agarose up a folate gradient using homemade plugins in ImageJ (17). Pseudopodia dynamics was quantified from DIC movies of cells migrating under agarose up a folate gradient by counting the number of frames during a pseudopodia extension manually using ImageJ. The Scar/WAVE complex dynamics; patch lifetime and patch frequency were calculated from the movies of cells migrating under agarose up a folate gradient using homemade plugins in ImageJ (Singh et al., 2021).

Graphs and statistical analyses were performed using Prism. To quantify the proportion of Scar/WAVE phosphorylation, the total intensity of all bands and the lowest band were determined using the Odyssey CLx Imaging system (LI-COR Biosciences, Lincoln, NE, United States). The percentage of phosphorylation was calculated by using the formula: % phosphorylation = (Total intensity−intensity of lowest band)*100/Total intensity. Statistical significance analyses were performed by non-parametric statistics, such as one-way ANOVA and Dunn’s multiple comparison tests, as described for each result. The experimental groups were tested for normal distribution using the Shapiro-walk test of Prism 7 software (Graphpad, Graphpad, San Diego, CA, United States). Sample sizes are provided in the figure legends. n refers to; independent experiments in western blotting, total number of cells from 3 independent experiments for cell speed, pseudopod dynamics, circularity, solidity and cell area.

## Results

### Scar phosphorylation is diminished in *pkbA-/pkgB-* cells

We recently showed that Scar/WAVE phosphorylation occurs after the activation of the complex and is to a large extent mediated by SepA kinase (Singh et al., 2020; Singh and Insall, 2020). However, in *sepA*- cells phosphorylation of the Scar/WAVE was not entirely eliminated, meaning that other kinases are also active. We therefore examined a range of mutants in known kinases implicated in cell migration.

**FIGURE 1:**
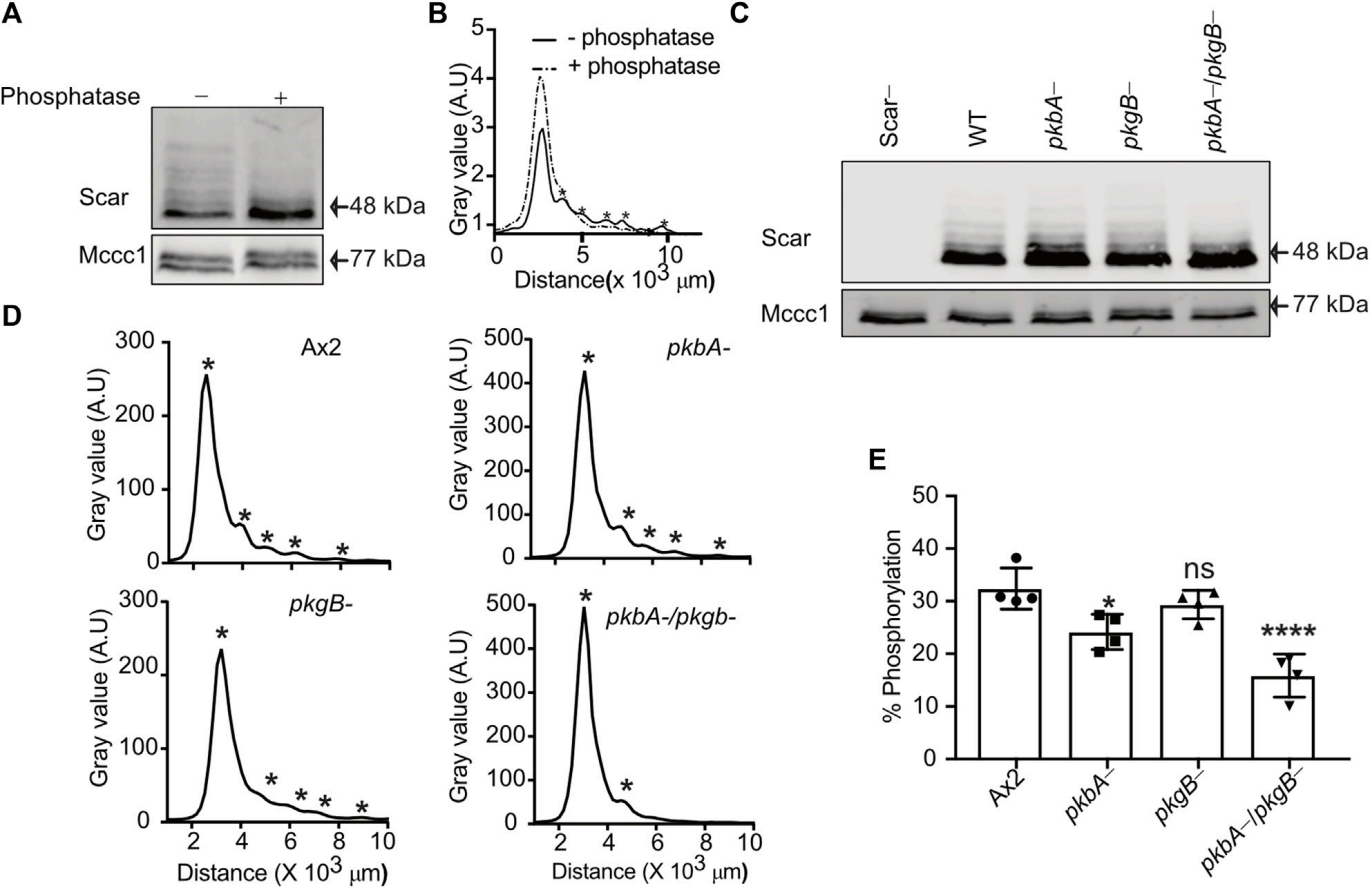
Phosphorylation of Scar is diminished in *Dictyostelium pkbA-/pkgB*-. **(A)** Assay of Scar phosphorylation. Ax2 cell lysates treated with phosphatase were analyzed by western blotting. **(B)** Density quantification shows multiple peaks (solid line) resolved into one (dotted line) after phosphatase treatment. Asterisks at the peaks represents Scar bands. **(C)** Assay of Scar phosphorylation in Ax2, *pkbA*-, *pkgB*- and *pkbA*-/*pkgB*- cells. Whole cell lysates were analysed by western blotting. **(D)** Density quantification shows flattening of multiple peaks (asterisks) in *pkbA*-, *pkgB*-, and loss in *pkbA-/pkgB*-. **(E)** Shows ratio of upper (more-phosphorylated) band to aggregate intensity of total Scar (bars show mean ± SD, *n* = 4 independent experiment, *p ≤ 0.05, ****p ≤ 0.0001, One-way Annova, Dunnett’s multiple comparison test). Mccc1 was used as loading control.

We recently established that the most effective way to measure the different phosphorylation states of Scar/WAVE uses western blots from low-bis acylamide gels. Phosphorylated proteins are visualised as a ladder of slower-migrating bands that are abolished by phosphatase treatment (Singh et al., 2020; Singh et al., 2021). We tested how far PKB and SGK contribute to Scar/WAVE phosphorylation in wild type (Ax2), *pkbA-*, *pkgB-* and *pkbA-/pkgB-* cells. In migrating *Dictyostelium* cells, multiple phosphorylated Scar bands can be resolved (Figure 1A), giving distinct peaks on the intensity plot (asterisks, Figure 1B). Lambda phosphatase treatment resolved all of the Scar phosphorylation bands into one single band and intensity peak (Figures 1A,B), confirming that the banding pattern is exclusively driven by different patterns of phosphorylation. The number of phosphorylated bands was unaltered in *pkbA-* and *pkgB-* cells, but interestingly, Scar phosphorylation was greatly diminished in the *pkbA-*/*pkgB-* double knockout cells (Figures 1C–E). We analyzed Scar band intensities by drawing a line across bands in ImageJ and plotted the values in graphs. Each peak on the graph represents one band of Scar (asterisks, Figure 1D). The intensity profile shows distinct multiple sharp peaks in Ax2, *pkbA-* cells and *pkgB-*, but only one in *pkbA-/pkgB-* cells (asterisks, Figure 1D). Quantification of the fraction of Scar in the upper bands shows a trend of reduction, which is 50% less in *pkbA-*/*pkgB-* cells (Figure 1E). This graph underestimates the total change in Scar phosphorylation, because the higher bands that are lost from *pkbA-*/*pkgB*- cells contain several phosphates (Singh et al., 2020). These results suggest that Scar/WAVE activation is altered in *pkbA-*/*pkgB-* cells but not in *pkbA*- or *pkgB*-.

### A role for PKB and PkgB in cell motility

**FIGURE 2:**
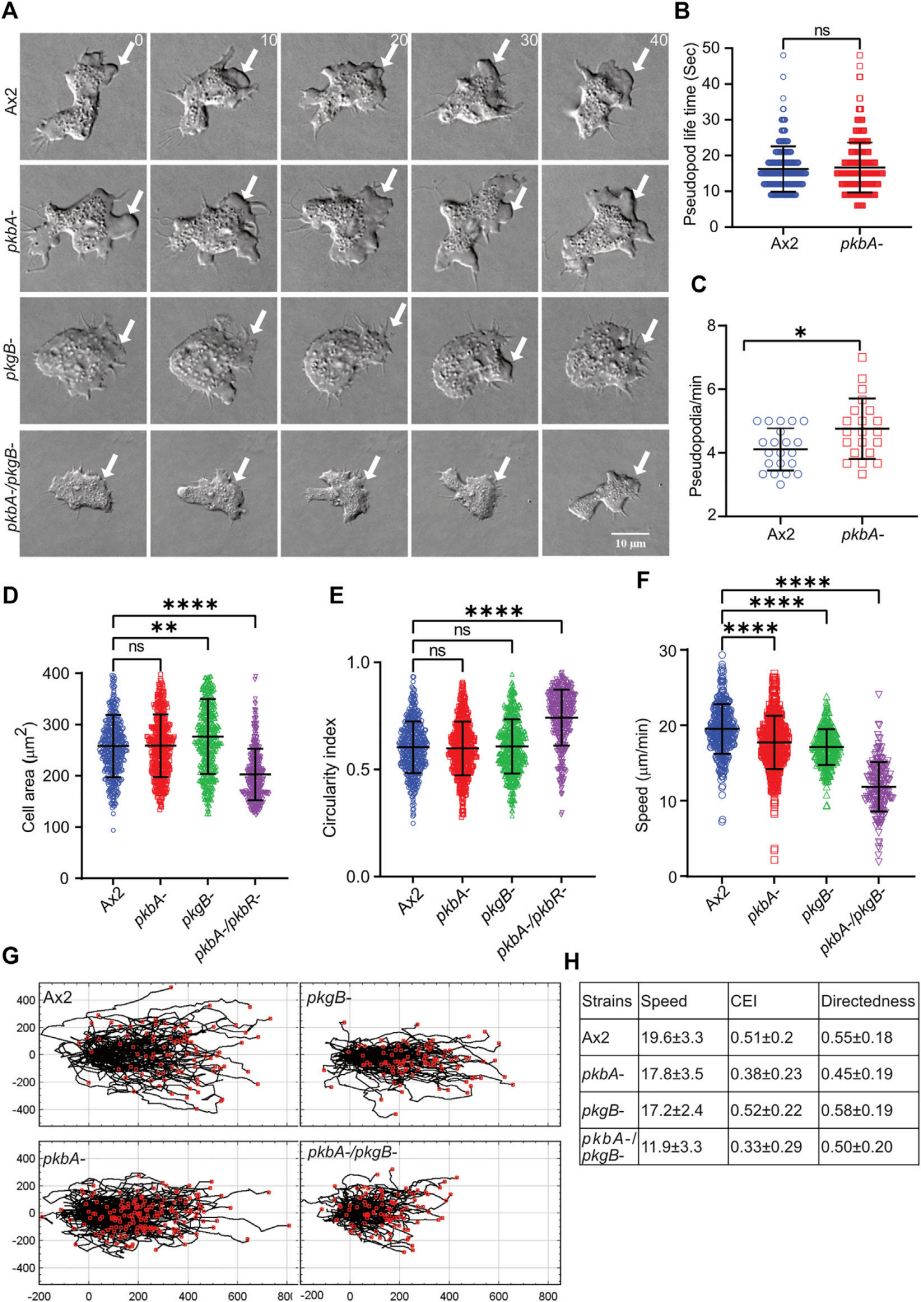
*pkgB* is required for efficient pseudopodia formation and cell migration. **(A)** Representative images of >20 cells showing pseudopodia formation in Ax2 and mutant cells. Ax2, *pkbA-*, *pkgB-* and *pkbA-/pkgB-* cells were allowed to migrate under agarose sensing a folate gradient and were imaged by DIC microscopy at a frame interval of 2 s (1f/2 s). Pseudopods are formed in Ax2 and single mutant *pkbA-*, but not in *pkgB-* and double mutant *pkbA*-/pkgB- (arrows). **(B–C)** Effect of *pkbA-* on pseudopods dynamics. Pseudopod lifetime and generation rate was measured from DIC videos using ImageJ from single frames, and generation rate calculated from the number of pseudopods lasting at least 2 frames. Lifetime of pseudopodia is equal in both Ax2 and *pkbA-* cells, but *pkbA-* generates pseudopods more frequently. (Mean ± SD, *n* > 20 cells, *n* > 25 cells from 3 experiments; *p ≤ 0.05, 1-way ANOVA, Mann-Whitney test). **(D,E)** Shows quantification of cell area and circularity index. Cell area is unchanged in Ax2 and *pkbA-*, increased in *pkgB-*, but decreased in *pkbA-*/*pkgB-*. Circularity index is only increased in *pkbA-/pkgB-*. (mean ± SD; *n* = ^416^Ax2, ^474^ *pkbA*-, ^458^ *pkgB*- and ^366^ *pkbA-/pkgB*- over 3 independent experiments, **p ≤ 0.01, ****p ≤ 0.0001, 1-way ANOVA, Dunn’s multiple comparison test). **(F)** Shows quantification of cell speed. Mutants migrate slower than Ax2 cells and (mean ± SD; *n* = ^361^Ax2, ^496^ *pkbA-*, ^298^ *pkgB-* and ^*200*^ *pkbA-/pkgB-* over 3 independent experiments, ****p ≤ 0.0001, One-way ANOVA, Dunn’s multiple comparison test). **(G)** Shows cell tracks of migrating cells. **(H)** Shows quantified values of speed, chemotactic efficiency index (CEI) and directedness of cells.

Phosphorylation of Scar/WAVE impacts cell motility and protrusion dynamics (Singh et al., 2020). To assess the role of PKB and PkgB in cell migration, we assayed the ability of Ax2, *pkbA-*, *pkgB-* and *pkbA-/pkgB-* cells to move up a folate gradient under agarose chemotaxis and examined their migratory phenotypes (Figure 2; Supplementary Videos S1–S3). Like Ax2, *pkbA-* cells formed pseudopodia and migrated efficiently. However, *pkgB-* and *pkgB-/pkbA-* cells do not form proper pseudopodia and migrated with the aid of thin, short-lived protrusions (arrows, Figure 2A; Supplementary Video S1). The formation of pseudopods in *pkbA-* cells was more frequent compared to Ax2, but once formed they lasted for a similar time (Figures 2B,C). Since *pkgB*- and *pkbA-/pkgB-* cells do not form normal pseudopods, their lifetime and frequency could not be quantified. Instead, we quantified cell spreading (cell area) and polarity (circularity and solidity). In both cases *pkgB-/pkbA-* mutants were substantially compromised, while *pkgB-* cells showed slight defects and *pkbA-* cells seemed unaffected (Figures 2D,E; Supplementary Figure S1). All kinase mutants appear to migrate slower than the Ax2 parental cells (Figure 2F), though the double mutant differs hugely. Directedness and chemotactic efficiency index are unaffected in all mutants (Figures 2G,H). The same is not true of shape, which is of course profoundly affected by the loss of normal pseudopod formation. The *pkbA -/pkgB-* double knockout has ∼20% smaller projected area and a substantially higher circularity and solidity (Figures 2D,E,G,H; Supplementary Figure S1). All together these results show that loss of both *pkbA* and *pkgB* impairs formation of pseudopodia, which results in defective cell motility, but has no obvious effect on guidance.

To confirm that the defects in motility and pseudopod generation are due to PKB and PkgB depletion, we expressed individual kinases in *pkgB-* and *pkbA-/pkgB-* cells and observed the rescue of pseudopods in migrating cells in an under-agarose folate chemotaxis assay (Supplementary Figures S2A,B, Supplementary Videos S2, S3). Rescued *pkgB-/pkgB* cells, somewhat unexpectedly, migrated with a slower speed than *pkgB-* with unaffected cell polarity (circularity and solidity) (Supplementary Figures S2C,D,E,F). This effect is possibly due to a dominant effect of overexpression of *pkgB* (Kamimura et al., 2008). Expression of either *pkbA* or *pkgB* in *pkbA-/pkgB-* cells substantially restores pseudopod formation, increases cell speed and polarity (Supplementary Figures S2B,C,D,E; Supplementary Video S3). This may be due to the overlap of substrates between the two kinases, meaning that overexpression of PKB may compensate for the lack of PkgB (Kamimura et al., 2008; Williams et al., 2019).

### Both PKB and PkgB are required for proper dynamics and spatiotemporal distribution of actin

**FIGURE 3:**
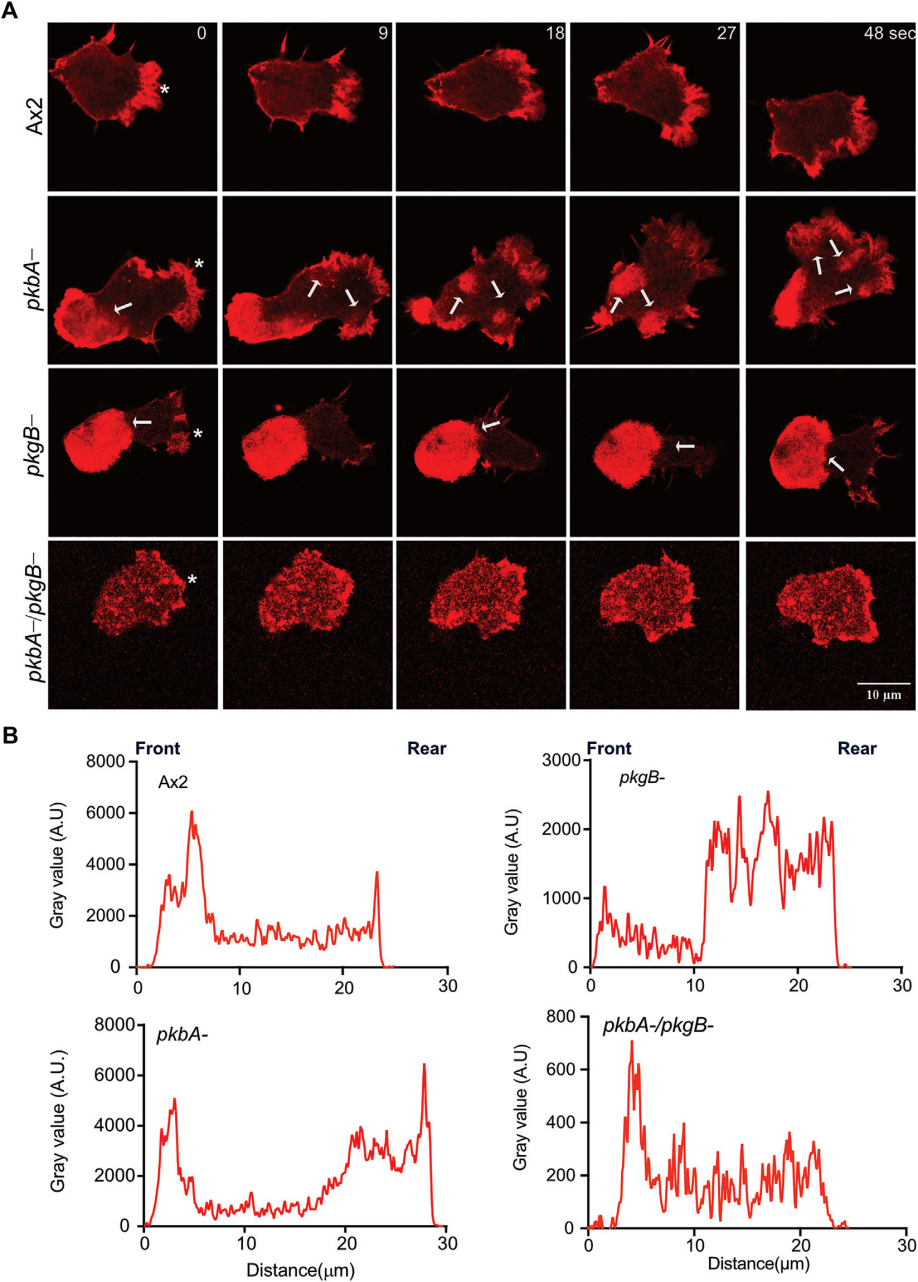
Actin localizes to Ax2 and *pkbA*- protrusions, but not in *pkgB*- and *pkbA*-/*pkgB*-. **(A)** Subcellular localization of F-actin (Red). Life-act-mRFPmars2 was expressed in Ax2, and mutant cells. Cells were allowed to migrate under agarose mediated by a folate gradient and imaged by AiryScan confocal microscopy at a frame interval of 3 s (1f/3 s). Ax2 and *pkbA*- cells show F-actin in pseudopodia (asterisk). F-actin accumulation is absent in *pkgB*- protrusions, and highly reduced in *pkbA*-/*pkgB*-. Static F-actin in the cell body is present in both *pkbA*- and *pkgB*-. **(B)** Quantification of the florescence intensities in cells. Using ImageJ, intensity profile plots were generated by drawing a straight line across the cell. The amount of F-actin is similar in Ax2 and *pkbA*-, but reduced in *pkgB*- and *pkbA*-/*pkgB*- cells, where F-actin is mostly localized in the cell body. Images are representative of ≥10 cells from 3 independent experiments.

Pseudopods are actin rich structures, which drive the movement of cells during migration. Cells deficient in *pkgB-* and *pkbA-/pkgB-* cells do not form proper pseudopodia (Figure 2A; Supplementary Video S1). This may be attributed to poor actin polymerization and distribution during cell migration (Kamimura et al., 2008; Liao et al., 2010; Srinivasan et al., 2013). To examine actin polymerization and dynamics in Ax2 and mutant cells, we expressed Lifeact-mRFPmars2 (F-actin marker). F-actin efficiently labels the pseudopods in migrating Ax2 and *pkbA-* cells (asterisk, Figure 3A). Unlike Ax2, *pkbA-* cells had a strong F-actin enrichment in pseudopods and in the uropod (arrows, Figure 3A; Supplementary Video S4). Both Ax2 and *pkbA-* cells had approximately similar F-actin intensities in their pseudopods (Figure 3B). However, a striking feature of polymerized actin in the pseudopods of *pkbA-* was the static nature of its actin structures, when pseudopodia were growing due to polymerization of new actin (arrows in *pkbA-* panel; Figure 3A; Supplementary Figure S3A; Supplementary Video S4). Approximately 32% of Ax2, 88% of *pkbA*-, 100% of *pkgB-* and 32% of *pkbA-/pkgB*- cells have F-actin accumulation in the rear or cell body (Supplementary Figures S3A,B). This may be due to poor pseudopodia detachment from the substratum or enhanced adhesion, which could be the reason for the slightly slower migration of *pkbA-* cells, and lack of actin reaching pseudopodia formation in *pkgB-* cells.

In contrast, in *pkgB-* cells, F-actin is mainly accumulated in the cell body, but not in protrusions (Figure 3A; Supplementary Video S4). Intensity plots confirmed that the protrusions of *pkgB-* have much reduced F-actin (Figure 3B). Impaired actin polymerization in the protrusions, and accumulation of F-actin in the cell body could explain the slight migratory defect in those cells (Figure 2D). Interestingly, little actin localization with much reduced intensity compared to Ax2 was observed at the cell periphery and front of *pkbA-/pkgB-* cells (Figures 3A,B; Supplementary Video S4). These results suggest that PkgB is crucial, but both PKB and PkgB are important for regulation of actin polymerization in pseudopods.

### The Scar/WAVE and Arp2/3 complexes activation is altered in *pkgB-* and *pkbA-*/*pkgB*-

Stability of the Scar/WAVE complex is important for its activity modulation (Ura et al., 2012; Davidson et al., 2013b). Hence, we investigated the influence of PKB and PkgB loss on the stability of the Scar/WAVE complex in Ax2 and mutant strains. We expressed HSPC300-eGFP in Ax2 and mutant cells and purified the complex using a GFP-TRAP pulldown. Western blot analysis for Pir121, Nap1, Scar, Abi and GFP showed stable complex formation in all cell lines (Supplementary Figure S4A).

**FIGURE 4:**
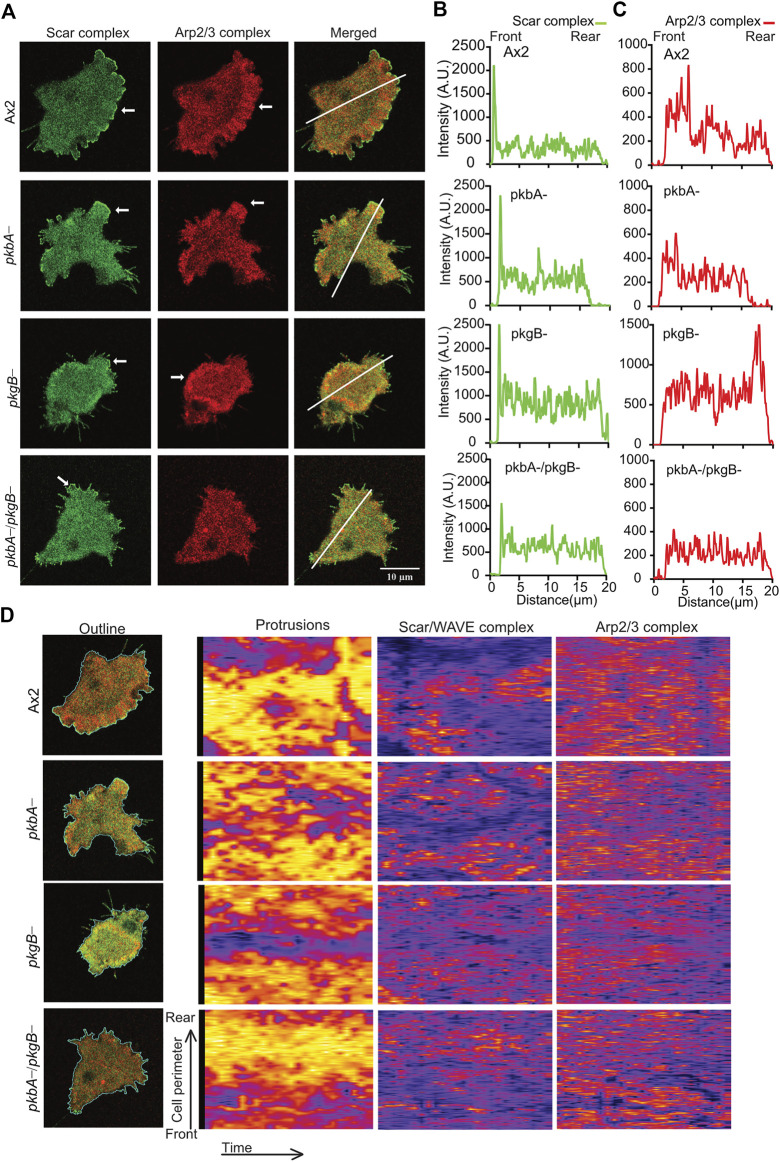
The recruitment of Scar/WAVE and Arp2/3 complexes in Ax2 and mutant cells. **(A)** Subcellular localization of the Scar/WAVE and the Arp2/3 complexes in pseudopods. Ax2, *pkbA*-, *pkgB*- and *pkbA-/pkgB*- cells were labelled with HSPC300-eGFP (Scar/WAVE complex) and mRFPmars2-ArpC4 (Arp2/3 complex) and allowed to migrate under agarose following a folate gradient and imaged by AiryScan confocal microscopy at a frame interval of 3 s (1f/3 s). The Scar/WAVE complex is localized to protrusions of pseudopodia of all cell types (arrows, panel 1). The Arp2/3 complex is enriched in the pseudopodia of Ax2 and *pkbA*- cells, in the cell body of *pkgB*-, but is lacking in the *pkbA-/pkgB*- cells (arrows, panel 2). **(B,C)** Quantification of the Scar/WAVE and Arp2/3 complex florescence intensities along the indicated line across the cell (panel 3 of A). **(D)** Quantitative assessment of the Scar/WAVE and Arp2/3 complex recruitment in cells. Panel 1; shows the outline of cells used for the generation of kymographs using ImageJ. Panel 2 represents protrusions, panel 3; the Scar/WAVE complex and panel 4; the Arp2/3 complex. Images are representative of ≥10 cells from 3 independent experiments.

The recruitment for Scar/WAVE complex, and the Arp2/3 complex activation it mediates, are pre-requisites for the polymerization of actin in pseudopods or lamellipods (Insall and Machesky, 2009; Krause and Gautreau, 2014). To measure the effects of *pkbA* and *pkgB* mutation on the recruitment of the Scar/WAVE and activation of the Arp2/3 complex, we co-expressed HSPC300-eGFP and ArpC4-mRFPmars2 (Veltman et al., 2012) in mutant strains and examined recruitment in migrating cells. The Scar/WAVE complex localizes in the protrusions of all strains with marked differences in the intensities, dynamics, and activity. The Scar/WAVE and Arp2/3 complexes colocalised in the protrusions of Ax2 (Figure 4A; Supplementary Video S5). The recruitment of both Scar and Arp2/3 complexes was compromised in the protrusion of *pkbA*-, *pkgB-* and *pkbA-*/*pkgB-* cells (Figures 4A–C; Supplementary Figures S4B,C; Supplementary Video S5). Both complexes accumulated substantially in the cell body of *pkgB-* cells (see *pkgB*- panel in Figures 4A–C; Supplementary Figure S4B; Supplementary Video S5). Intensity plots of the Scar/WAVE and Arp2/3 complexes indicate similar intensity in protrusions of all cells, but the mutants have more cytosolic accumulation (Figures 4A,B; Supplementary Video S5).

**FIGURE 5:**
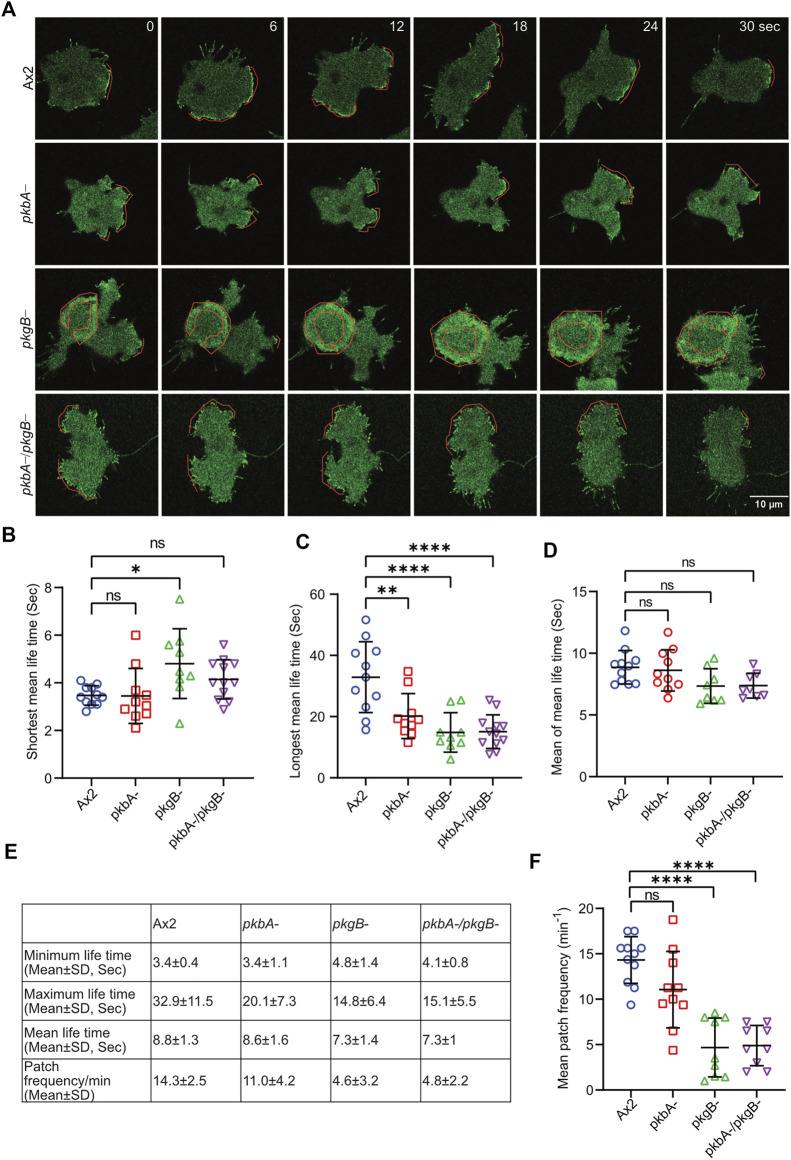
The recruitment of the Scar/WAVE complex in Ax2 and mutant cells. Subcellular localization of the Scar/WAVE complex in protrusions. **(A)** Ax2, *pkbA*-, *pkgB*- and *pkbA-/pkgB*- cells were labelled with HSPC300-eGFP (Scar/WAVE complex) and allowed to migrate under agarose following a folate gradient and imaged by AiryScan confocal microscopy at a frame interval of 3 s (1f/3 s). The Scar/WAVE complex is localized to protrusions of pseudopodia of all cell types (dotted line). **(B–D)** Quantification of lifetime to Scar/WAVE recruitment. The lifetime of the Scar/WAVE complex accumulation was quantified using homemade ImageJ plugin. Mean lifetimes of shortest **(B)**, longest **(C)** and mean **(D)** Scar/WAVE duration were plotted. (*n* > 9 cells over three experiments, mean ± SD, ns = non-significant *p ≤ 0.05, **p ≤ 0.01, ***p ≤ 0.001, One-way ANOVA, Dunn’s multiple comparison test). **(E)** Scar/WAVE lifetimes in protrusions of cell lines. **(F)** Scar/WAVE recruitment frequency in the protrusions of cell-lines.

The frequency and lifetime of Scar/WAVE is in general highly dynamic. In the mutants, recruitment seemed transient and oscillatory. To quantify the differences in the lifetime of the Scar/WAVE and Arp2/3 complexes in our cells, we generated whole-boundary kymographs and intensity graphs using a custom-made ImageJ plugin. In all cell lines, protrusion (Figure 4D, protrusions column) is highly correlated with Scar/WAVE recruitment (compare Figure 4D, Scar/WAVE column), with protrusions centered on areas rich in Scar/WAVE. WT Ax2 cells have large, long-lasting pseudopods centred on longer-lasting Scar patches (dotted line; Figures 5A–D). The lifetimes of these patches are reduced in *pkbA-* and reduced further still in both *pkgB-* and double mutant cells (Figures 5B–E). In *pkgB*- cells, Scar mostly accumulated in the periphery of the cell body, and in the *pkbA-/pkgB*-, Scar accumulation was observed mostly as puncta in protrusions (dotted line; Figure 5A).

The same correlation holds true in *pkbA-* cells, though they have a broader spread of shorter-lived Scar/WAVE flashes (Figures 4D, 5B–E–5E). In both *pkgB-* and *pkbA-*/*pkgB-* cells, Scar/WAVE and Arp2/3 complex flashes were transient (compare Arp2/3 column of Figure 4D with Supplementary Figure S4B). Moreover, Scar/WAVE recruitment frequency was highly reduced in mutant cell-lines, compared to Ax2 (Figures 5E,F). This suggests that both PKB and PkgB are required for the proper activation of the Scar/WAVE complex and coupling from active Scar/WAVE to the Arp2/3 complex.

## Discussion

We report that PKB and PkgB regulate Scar/WAVE phosphorylation and pseudopod formation in *Dictyostelium*. We have shown that Scar/WAVE phosphorylation is strongly reduced in *pkbA-/pkgB-* cells. Single mutants were largely unaffected, and PKB and PkgB, both share a conserved kinase motif, suggesting redundancy in their substrates. The classical Akt/Sgk motif RXRXXS^∗^/T^∗^ is not found within the serine residues that have been found to be important for Scar phosphorylation (Singh et al., 2020), nor is any member of the Scar/WAVE complex among the known targets of these enzymes. It therefore seems likely that PKB and PkgB control Scar phosphorylation through other, intermediate kinases. This assumption is supported by phospho-proteomics data published for *pkbA-*, *pkgB-* and double mutant which include known kinases as KrsB and Pats1 (Williams et al., 2019). However, there is a strong possibility that both PKB and PkgB are in some way involved in the regulation of SepA which has been previously reported to be essential for Scar/WAVE phosphorylation. The *sepA* knockout showed a similar level of reduction in phosphorylation, making a connection between the kinases likely (Singh et al., 2020). How this crosstalk between the different kinases is mediated, will need to be further investigated in the future.

We used folate chemotaxis to assay the morphological and cytoskeletal changes caused by loss of *pkbA* and *pkgB*. These changes appear to be driven by changes in the phosphorylation state of Scar/WAVE. This is likely to be true for other chemoattractants - cAMP and folate induce an identical set of substrate phosphorylation and operate through common pathways (Liao et al., 2010; Srinivasan et al., 2013), but we have focussed on folate because loss of PKB affects development. While common signaling networks are involved in the response to both chemoattractants, differences have been observed before when comparing folate to cAMP chemotaxis in the same mutant cell line (Nichols et al., 2019). cAMP chemotaxis does not only depend on the actin cytoskeleton but also on cell differentiation and the correct expression of developmental markers to make cells able to respond efficiently to cAMP.

Previous studies have reported that both kinases are important for cAMP chemotaxis (Meili et al., 1999; Meili et al., 2000; Kamimura et al., 2008; Kamimura and Devreotes, 2010). In contrast, this work reveals that both PKB and PkgB are dispensable for chemotaxis to folate but important for pseudopod formation and cell speed. This is less likely to represent changes in mechanism, and more likely a complication of developmental changes in *pkbA* and *pkgB* mutants. There are also assay-specific differences in the way cells experience the gradient of chemoattractant. We view cells in a self-generated gradient when performing chemotaxis under agarose, because this gives optimal conditions for visualising Scar/WAVE complex recruitment during pseudopod formation. The behavior of cells is different when they respond to a chemoattractant that is released from micropipettes.

We know that mechanism of chemotaxis does not principally work through initiation of new protrusions, but rather through changes to pseudopod dynamics (Andrew and Insall, 2007; Bosgraaf and Van Haastert, 2009). Pseudopods control chemotactic steering (Andrew and Insall, 2007; Bosgraaf and Van Haastert, 2009), persistence, polarity, cell shape and motility (Fort et al., 2018). However, in our assays *pkgB-* and *pkbA-*/*pkgB-* cells move towards folate sources without forming proper pseudopods, which raises the possibility of pseudopodia independent chemotaxis, using other transient protrusions. The defects in pseudopod formation in *pkgB-* is more severe than in *pkbA-.* This might be explained by a higher level of substrate phosphorylation by PkgB compared to PKB (Kamimura et al., 2008).

As the loss of pseudopod formation in *pkbA-/pkgB*- cells can be rescued by extrachromosomal expression of either *pkbA* or *pkgB*. This is because both kinase share substrates and over-expression of either kinase has been shown to rescue phenotypes of *pkbA-/pkgB*- cells (Meili et al., 2000; Williams et al., 2019).

To generate and extend pseudopodia, cell substrate adhesion is essential (Ladoux and Nicolas, 2012). The defects observed in *pkgB-* and *pkbA-/pkgB-* may be due to the inability of pseudopods to attach with substratum. Talin integrates, adhesion molecules like integrins (homolog of SibA&B in *Dictyostelium*) to the actin cytoskeleton (Niewohner et al., 1997; Simson et al., 1998). In *Dictyostelium* Talin B has been identified as one of the substrates of PKB and PkgB (Chen et al., 2017) and cells lacking TalinB have severe adhesion and motility defects (Tsujioka et al., 1999; Tsujioka et al., 2004). We have also reported that adhesion stimulates activation and phosphorylation of Scar/WAVE in pseudopods of migrating cells (Singh et al., 2020; Singh and Insall, 2020).

Additionally, we observed impaired activation of the Scar/WAVE and Arp2/3 complexes in the protrusions of *pkgB*- and *pkbA-/pkgB*- cells. This leads to a strongly reduced level of F-actin at the leading edge of the cell, due to the disrupted interaction between both complexes (Scar/WAVE at the front, Arp2/3 at rear in *pkgB-* or barely expressed in the double mutant). The hyperaccumulation of the Scar/WAVE, Arp2/3 complexes and F-actin in cell body indicates that PkgB is involved in the recruitment of the Scar/WAVE complex in the pseudopodia. The eventual destination of the Scar/WAVE complex is exceptionally complicated, being affected by positive feedback, retrograde actin flow, and a poorly-understood, activation-dependent degradation mechanism. We note that in *pkgB*- cells, Scar mostly accumulated in the periphery of the cell body, whereas in the *pkbA*-/*pkgB*-, it was observed mostly as puncta in protrusions (Figure 5A). It is not yet clear what causes this difference.

The undermined Scar/WAVE and Arp2/3 complexes recruitment in *pkbA*-, *pkgB-* and *pkbA-/pkgB-* hints that both PKB and PkgB seem important for Arp2/3 complex localization and activation.

Overall, this study finds that PkgB independently or together with PKB regulates pseudopodia formation and cell migration *via* the Scar/WAVE and Arp2/3 complexes and raises multiple questions regarding regulation of cell motility.

## Data Availability

All the raw data for the figures and videos used in this study can be requested from RI (Robert.Insall@glasgow.ac.uk) and SS (Shashi.Singh@glasgow.ac.uk).
