# Supplementary material for: AKT and SGK kinases regulate cell migration by altering Scar/WAVE complex activation and Arp2/3 complex recruitment
Source: Front Mol Biosci. 2022 Aug 29;9:965921. doi: 10.3389/fmolb.2022.965921 (PMC9466652; doi:10.3389/fmolb.2022.965921)
Supplement: Supplementary file 2 [file DataSheet1.pdf]

Supplementary materials

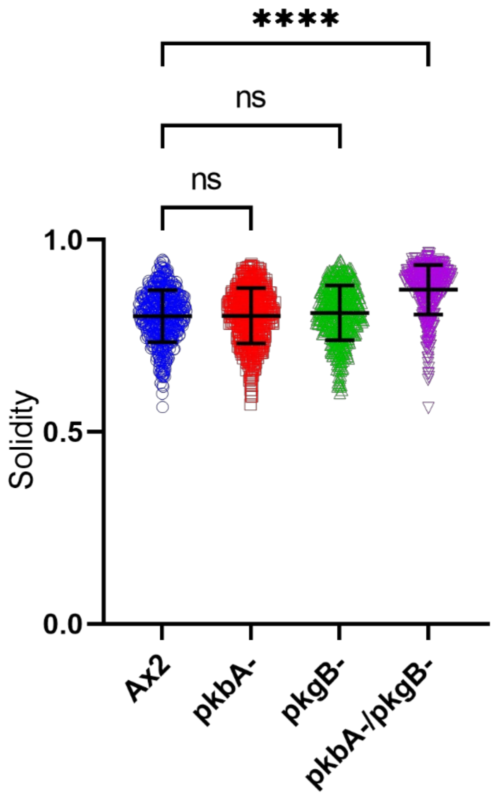

Figure S1. Change in cell solidity in Ax2, and *pkbA-*, *pkgB-*, and *pkbA-*/*pkgB-* cells. Ratio of total cell area and convex cell areas was calculated using a custom-made Image J plugin. (mean ± SD; n = ^{416}Ax2, ^{474}*pkbA-*, ^{458}*pkgB-* and ^{366}*pkbA-*/*pkgB-* over 3 independent experiments, ****p ≤ 0.0001, 1-way ANOVA, Dunn's multiple comparison test).

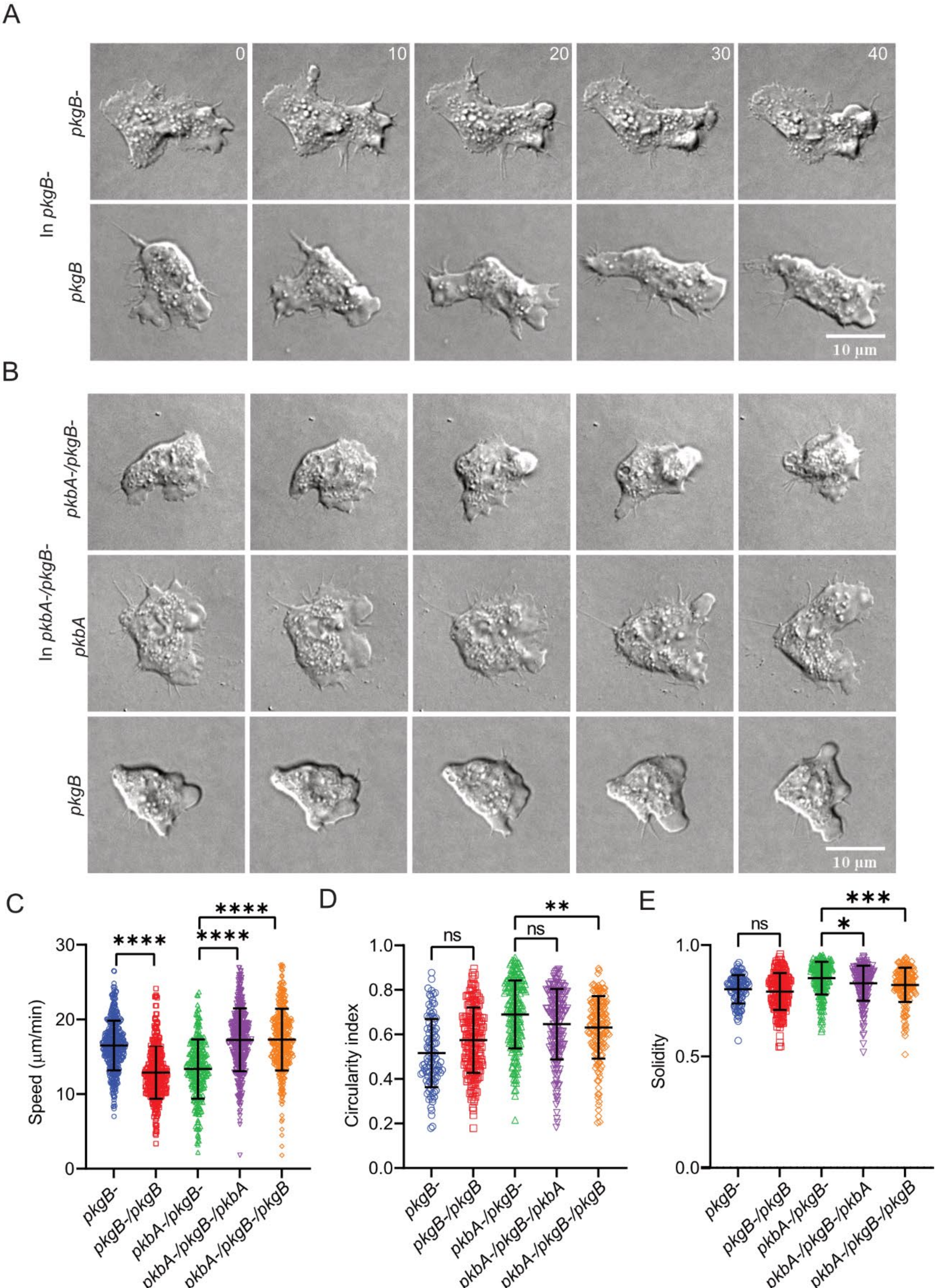

F

Cells	Speed(µm/min; mean±SD)	Circularity index	Solidity
pkgB-	16.62±3.3	0.51±0.15	0.8±0.06
pkgB-/pkgB	12.9±3.5	0.57±0.14	0.79±0.08
pkbA-/pkgB-	13.3±3.9	0.68±0.15	0.85±0.07
pkbA-/pkgB-/pkbA-	17.2±4.2	0.64+0.15	0.82±0.07
pkbA-/pkgB-/pkgB-	17.3±4.1	0.63±0.14	0.82±0.07

Figure S2. Rescue of pseudopodia formation in *pkgB-* and *pkbA-/pkgB-*. (A) Extrachromosomal expression of *pkgB* rescues pseudopodia formation in *pkgB*- cells. (B) Extrachromosomal expression of *pkbA* (panel 2) and *pkgB* (panel 3) rescues pseudopodia formation in *pkbA-/pkgB*- cells. (C) Quantification of speed of *pkgB*-, *pkbA-/pkgB*- and rescued cells. (mean ± SD; n >300 cells over 3 independent experiments, ****p ≤ 0.0001, 1-way ANOVA, Dunn's multiple comparison test). (D&E) Quantification of circularity index and solidity of *pkgB*-, *pkbA*-/*pkgB*- and rescued cells, (mean ± SD; n>50 cells over 3 independent experiments, *p ≤ 0.05, **p ≤ 0.01, ***p ≤ 0.001, 1-way ANOVA, Dunn's multiple comparison test). (F) Quantitative measurement of speed, circularity index and solidity.

A

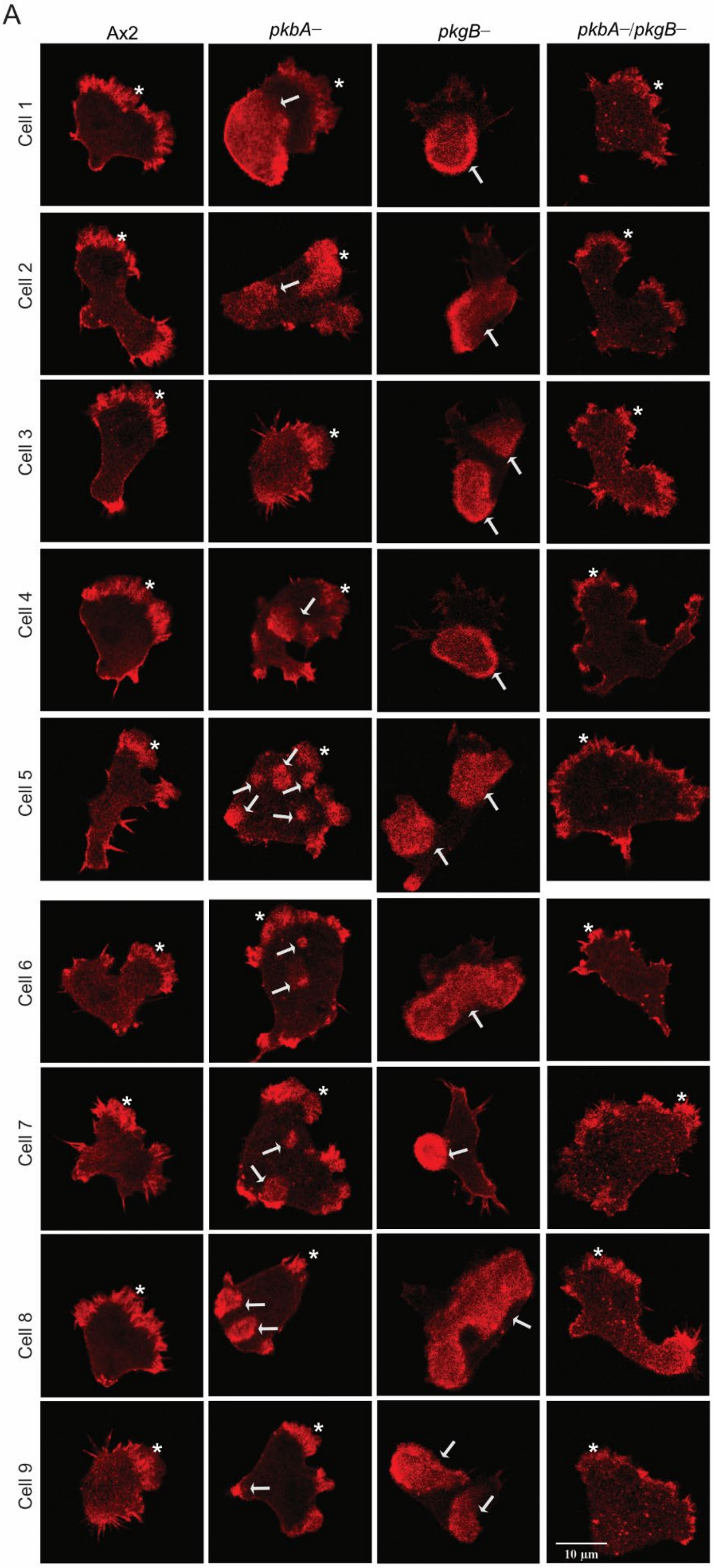

B

Cells	% of cells
Ax2 (n=19)	32
pkbA- (n=17)	88
pkgB- (n=12)	100
pkbA-/ pkgB- (n=19)	32

Figure S3: Comparison of F-Actin distribution in multiple cells of Ax2 and mutant cells by AiryScan confocal microscopy. (A) Panel of cells showing F-actin accumulation in protrusions, cell body and rear. Life-act-mRFPmars2 was expressed in Ax2, and mutant cells. Cells were allowed to migrate under agarose mediated by a folate gradient and imaged by AiryScan confocal microscopy at a frame interval of 3 seconds (1f/3s). Ax2 and *pkbA*- cells show F-actin in pseudopodia (asterisks). F-actin accumulation is absent in *pkgB*- protrusions, and highly reduced in *pkbA*-/*pkgB*- cells (asterisks). Static F-actin in the cell body is mainly present in both *pkbA*- and *pkgB*- cells (arrows). (B) Percentage of cells with F-actin accumulation at the rear or cell body. n= number of cells ^{19}Ax2, ^{17}*pkbA*-, 12*pkgB*- and ^{19}*pkbA*-/*pkgB*-.

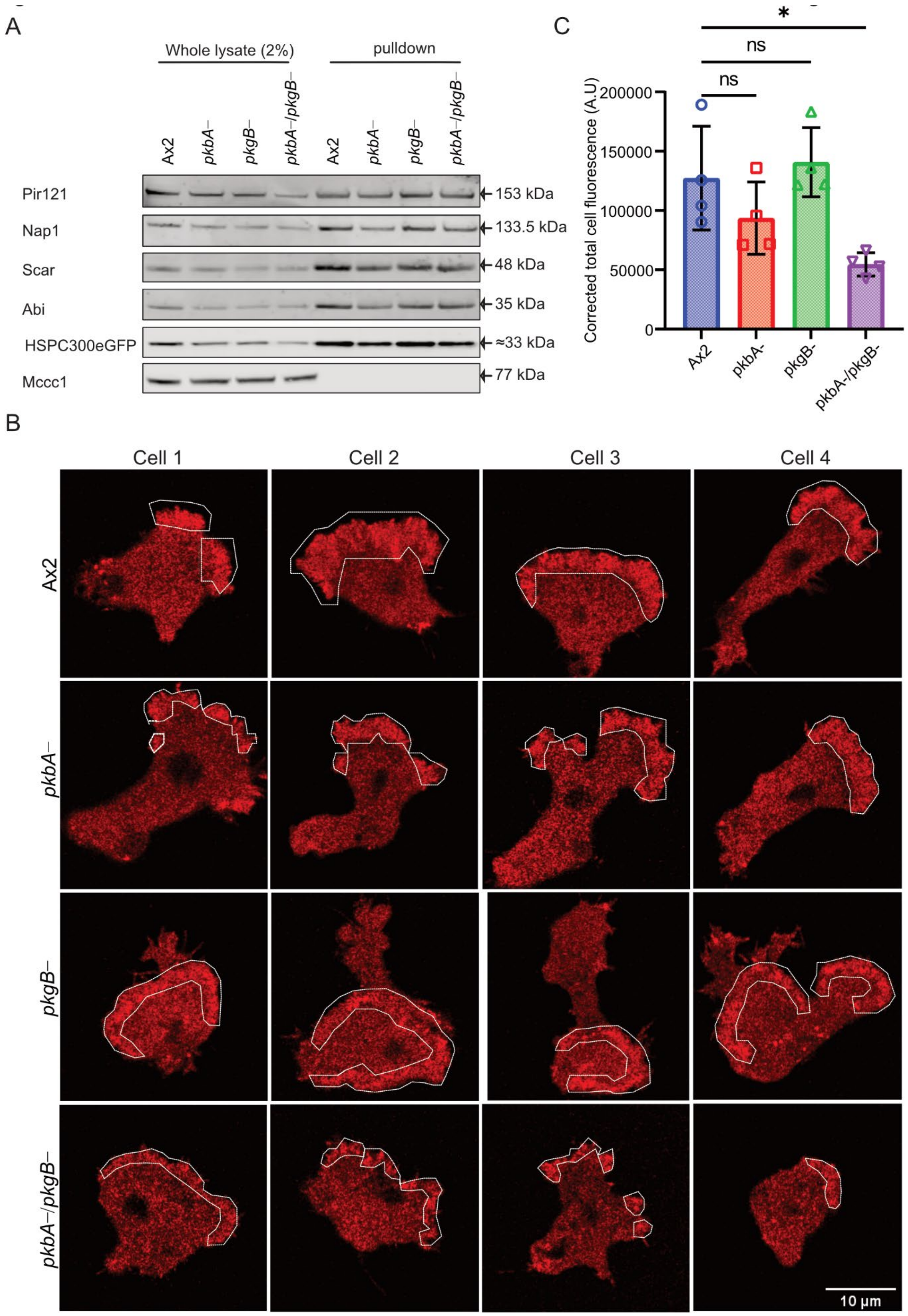

A
Whole lysate (2%)
pulldown
Ax2
pkbA–
pkgB–
pkbA–/pkgB–
Ax2
pkbA–
pkgB–
pkbA–/pkgB–
Pir121
Nap1
Scar
Abi
HSPC300eGFP
Mccc1
153 kDa
133.5 kDa
48 kDa
35 kDa
≈33 kDa
77 kDa
C
*
ns
ns
Corrected total cell fluorescence (A.U)
200000
150000
100000
50000
0
Ax2
pkbA-
pkgB-
pkbA-/pkgB-
B
Cell 1
Cell 2
Cell 3
Cell 4
Ax2
pkbA–
pkgB–
pkbA–/pkgB–
10 µm

Figure S4: Stable Scar/WAVE complex formation in Ax2 and mutants. (A)The Scar/WAVE complex was purified from cells expressing HSPC300-eGFP by GFP trap. Whole cell lysate and pulldown samples were analysed for the expression of, Pir121, Nap, Scar, Abi, 1 and GFP by western blotting. Mccc1 was used as loading control. This experiment was repeated thrice. (B) Recruitment of Arp2/3 complex in cell protrusions of multiple cells over 3 experiments. Ax2, *pkbA-*, *pkgB-* and *pkbA-/pkgB-* cells were labelled with mRFPmars2-ArpC4 (Arp2/3 complex), allowed to migrate under agarose following a folate gradient and imaged by AiryScan confocal microscopy at a frame interval of 3 seconds (1f/3s). The Arp2/3 complex is enriched broadly in the pseudopodia of Ax2 and *pkbA-* cells, in the cell body of *pkgB-*, but is highly reduced in the *pkbA-/pkgB-* cells (dotted lines). (C) Integrated fluorescent intensities of the Arp2/3 complex were calculated using image and normalized to cell area is represented. Reduced Arp2/3 complex intensity in the protrusions of *pkbA-* and *pkbA-/pkgB-* cells. Mean and SD, n=4, , *p ≤ 0.05.

Video legends

Video S1. Pseudopodia formation in Ax2, *pkbA-*, *pkgB-* and *pkbA-/pkgB-* cells during directed migration. All cells were allowed to migrate under agarose chasing a folate gradient and observed by DIC microscopy. Filmed at 1 frame/2 seconds, movie shows 10 frames/second.

Video S2. Rescue of pseudopodia in *pkgB-* by extrachromosomal expression of *pkgB*. *pkgB-* cells expressing pkgB were allowed to migrate under agarose up a folate gradient and observed by DIC microscopy. Filmed at 1 frame/2 seconds, movie shows 10 frames/second.

Video S3. Rescue of pseudopodia in *pkbA-/pkgB-* by extrachromosomal expression of *pkbA* or *pkgB*. *pkbA-/pkgB-* cells expressing *pkbA* or *pkgB* were allowed to migrate under agarose up a folate gradient and observed by DIC microscopy. Filmed at 1 frame/2 seconds, movie shows 10 frames/second.

Video S4. Localization of F-actin in Ax2 and mutant cells. Cells expressing Lifeact-mRFPmars2 were allowed to migrate under agarose up a folate gradient and observed by AiryScan confocal microscopy. **Filmed at 1 frame/3 seconds, movie shows 10 frames/second.**

Video S5. Recruitment of the Scar/WAVE and Arp2/3 complexes in Ax2 and mutant cells. Cells expressing HSPC300-eGFP and mRFPmars2-ArpC4 were allowed to migrate under agarose towards a folate gradient and observed by AiryScan confocal microscopy. Filmed at 1 frame/3 seconds, movie shows 10 frames/second.
